# Long-Term Clinical Outcomes and Real-World Effectiveness of Tirzepatide in Adults With Overweight or Obesity: A Systematic Review

**DOI:** 10.7759/cureus.114962

**Published:** 2026-08-22

**Authors:** Aliyu O Olaniyi, Khyathi Krishna Gogineni, Happiness Olaniyi, Hamza Sabir, Gloria Agbonrofo Eboigbe, Susuti R Yerima, Nazeem Gabriels

**Affiliations:** 1 Geriatrics, Stepping Hill Hospital, Stockport, GBR; 2 Stroke Medicine, Stepping Hill Hospital, Stockport, GBR; 3 Nursing, Barton Brook Care Home, Manchester, GBR; 4 Acute Medical Unit, Stepping Hill Hospital, Stockport, GBR; 5 Internal Medicine, Stockport NHS Foundation Trust, Stockport, GBR; 6 Hospital Services, Federal Ministry of Health and Social Welfare, Abuja, NGA; 7 Emergency Medicine, Stockport NHS Foundation Trust, Stockport, GBR

**Keywords:** cardiometabolic outcomes, gip, glp-1 receptor agonist, obesity, overweight, real-world evidence, systematic review, tirzepatide, weight loss, weight maintenance

## Abstract

Obesity is a chronic, progressive, and relapsing disease associated with substantial morbidity, premature mortality, and increased healthcare utilisation. Although lifestyle modification remains the cornerstone of management, long-term weight-loss maintenance is challenging. Tirzepatide, a once-weekly dual glucose-dependent insulinotropic polypeptide (GIP) and glucagon-like peptide-1 (GLP-1) receptor agonist, has demonstrated substantial weight-loss efficacy, but its long-term clinical outcomes have not been comprehensively synthesised.

A systematic search of Ovid MEDLINE ALL, Embase, the Cochrane Central Register of Controlled Trials (CENTRAL), and Cumulative Index to Nursing and Allied Health Literature (CINAHL) was conducted from database inception to June 30, 2026. Eligible studies included Phase III randomised controlled trials, randomised withdrawal trials, long-term extension studies, prespecified secondary analyses reporting unique outcomes, and real-world observational studies. Two reviewers independently screened studies, extracted data, and assessed methodological quality using the Cochrane Risk of Bias 2 (RoB 2) and Risk Of Bias In Non-randomized Studies of Interventions (ROBINS-I) tools. Owing to substantial clinical and methodological heterogeneity, findings were synthesised narratively in accordance with the Synthesis Without Meta-analysis (SWiM) guidelines.

Twenty-six publications representing 11 independent parent studies met the inclusion criteria, comprising eight Phase III randomised controlled trials, one long-term extension study, two real-world observational cohort studies, and 15 prespecified secondary analyses. Tirzepatide consistently produced substantial, dose-dependent, and durable weight reduction, with mean weight loss approaching 21% after 72 weeks. Continued therapy maintained weight loss, whereas treatment discontinuation resulted in progressive weight regain. Tirzepatide also improved cardiometabolic risk factors, body composition, physical functioning, and health-related quality of life. Gastrointestinal adverse events were the most common and were generally mild to moderate. Real-world studies demonstrated effectiveness and safety comparable to those observed in randomised clinical trials.

Current evidence indicates that tirzepatide provides durable weight reduction and broad cardiometabolic benefits in adults with overweight or obesity. Sustained treatment is important for maintaining these benefits, while additional long-term studies are needed to evaluate treatment persistence, cardiovascular outcomes, comparative effectiveness, and long-term safety across diverse populations.

## Introduction and background

Obesity is a chronic, multifactorial disease that has reached epidemic proportions globally. It is a leading cause of preventable morbidity, premature mortality, and escalating healthcare expenditures [1,2]. According to the World Health Organisation (WHO), over one billion individuals are affected by obesity, with prevalence increasing across all age groups and regions [2]. Obesity elevates the risk of numerous chronic conditions, including type 2 diabetes, hypertension, dyslipidaemia, cardiovascular disease, sleep apnoea, non-alcoholic fatty liver disease, chronic kidney disease, osteoarthritis, various cancers, and reduced life expectancy [1,3]. Furthermore, obesity imposes a substantial economic burden, resulting in increased healthcare utilisation, greater disability, diminished work productivity, and lower quality of life [4].

Current international guidelines now recognise obesity as a chronic disease that needs comprehensive, long-term management, not just short-term weight-loss efforts. Groups such as the European Association for the Study of Obesity (EASO), the American Association of Clinical Endocrinology (AACE), the American Diabetes Association (ADA), and an international consensus led by Wadden and colleagues recommend an evidence-based approach that combines behavioural changes, effective medications, and, when needed, metabolic surgery [5-7]. These recommendations reflect a better understanding that obesity is caused by complex neuroendocrine, metabolic, environmental, and behavioural factors that make it difficult to lose weight and easy to regain it after weight loss [5-8].

Dietary modification, increased physical activity, and behavioural therapy remain the primary strategies for obesity management; however, many individuals experience difficulty achieving and sustaining clinically significant weight loss [5-8]. Following weight reduction, physiological adaptations such as increased appetite, alterations in gut hormone levels, decreased resting energy expenditure, and changes in central energy regulation frequently promote weight regain, thereby limiting the long-term effectiveness of lifestyle interventions alone [8,9]. Consequently, long-term pharmacotherapy has become increasingly important for certain individuals with obesity.

Recent pharmacological advancements, particularly in incretin-based therapies, have transformed obesity treatment. Tirzepatide is the first once-weekly agent that targets both glucose-dependent insulinotropic polypeptide (GIP) and glucagon-like peptide-1 (GLP-1) receptors for long-term weight management. In contrast to agents that selectively target GLP-1, tirzepatide activates both receptors, thereby modulating appetite, enhancing satiety, delaying gastric emptying, improving insulin secretion, reducing glucagon levels, increasing insulin sensitivity, influencing lipid metabolism, and regulating energy balance [10-12]. Emerging evidence indicates that dual receptor agonism enhances appetite regulation, metabolic flexibility, and fat loss to a greater extent than GLP-1 receptor agonism alone, accounting for the robust weight reduction observed in clinical trials [12]. Initially developed for type 2 diabetes, tirzepatide has demonstrated substantial efficacy in promoting weight loss among adults with overweight or obesity, resulting in its approval for long-term weight management in multiple countries [7].

The SURMOUNT clinical trial program has demonstrated that tirzepatide induces significant and clinically meaningful weight loss across diverse populations, including adults with obesity (with or without diabetes), individuals who have previously attempted intensive lifestyle interventions, those with obesity-related sleep apnoea, and Asian cohorts [6,13-18]. Recent research has expanded beyond the primary Phase III trials to encompass long-term follow-up studies, withdrawal trials, secondary analyses, and real-world investigations. Collectively, these studies have advanced understanding of the durability of weight loss, outcomes following treatment discontinuation, diabetes prevention, changes in body composition, insulin sensitivity, β-cell function, blood pressure, cardiometabolic risk, quality of life, mental health safety, nutritional status, and real-world effectiveness [19-21].

Despite recent advances, significant knowledge gaps remain. Most systematic reviews were conducted prior to the completion of the full SURMOUNT program and therefore included only the earliest Phase III trials. Consequently, earlier reviews primarily addressed short-term weight loss and safety, with limited attention to long-term weight maintenance, weight regain following treatment cessation, diabetes prevention, patient-reported outcomes, cardiometabolic effects, pharmacological mechanisms, and real-world outcomes [19-21]. Additionally, numerous recent secondary analyses have reported on distinct cardiometabolic, nutritional, mental health, and functional outcomes, yet these findings have not been comprehensively synthesised, despite their relevance to the primary trial results [19-21].

This systematic review aims to synthesise all available evidence regarding the efficacy and safety of tirzepatide for long-term use, weight-loss maintenance, weight regain following treatment discontinuation, cardiometabolic outcomes, patient-reported outcomes, and real-world effectiveness in adults with overweight or obesity. By incorporating data from Phase III trials, long-term follow-up studies, withdrawal trials, secondary analyses, and real-world investigations, this review provides a comprehensive and up-to-date assessment of tirzepatide’s role in long-term obesity management. This work has not been previously presented as a conference abstract, poster, oral presentation, or at any scientific meeting.

## Review

Materials and methods

This systematic review evaluated the long-term efficacy, safety, weight-loss maintenance, weight regain following treatment discontinuation, cardiometabolic outcomes, patient-reported outcomes, and real-world effectiveness of tirzepatide in adults with overweight or obesity. The review was conducted in accordance with the Cochrane Handbook for Systematic Reviews of Interventions and is reported in accordance with the Preferred Reporting Items for Systematic Reviews and Meta-Analyses (PRISMA) 2020 guidelines [22,23].

A comprehensive systematic search was conducted from database inception to June 30, 2026, in four electronic bibliographic databases: Ovid MEDLINE ALL, Embase, the Cochrane Central Register of Controlled Trials (CENTRAL), and Cumulative Index to Nursing and Allied Health Literature (CINAHL) Ultimate. Ovid MEDLINE ALL was used to identify biomedical and clinical literature; Embase to capture pharmacological and European biomedical studies; Cochrane CENTRAL to identify randomised and quasi-randomised controlled trials; and CINAHL Ultimate to retrieve nursing and allied health literature. To maximise study identification, the reference lists of all included studies and relevant systematic reviews were also manually searched for additional eligible publications.

Study eligibility was determined using the Population, Intervention, Comparator, Outcomes, and Study design (PICOS) framework recommended by the Cochrane Handbook [22]. Eligible studies enrolled adults aged 18 years or older with overweight (body mass index (BMI) ≥27 kg/m²) and at least one weight-related comorbidity, or obesity (BMI ≥30 kg/m²). Studies involving participants with obesity and prediabetes, type 2 diabetes mellitus (T2DM), or obesity-related obstructive sleep apnoea (OSA) were included, provided overweight or obesity was the primary indication for tirzepatide treatment. Studies involving children, pregnant women, or participants receiving tirzepatide primarily for conditions unrelated to overweight or obesity were excluded.

The intervention of interest was once-weekly subcutaneous tirzepatide administered at maintenance doses of 5 mg, 10 mg, or 15 mg, either alone or alongside lifestyle intervention. Comparator groups included placebo, lifestyle intervention alone, standard care, active pharmacological comparators (e.g., semaglutide), treatment continuation versus withdrawal, or no comparator in real-world observational studies.

The primary outcomes were percentage and absolute changes in body weight, weight-loss maintenance during continued treatment, and weight regain following treatment discontinuation. Secondary outcomes included changes in BMI, waist circumference, body composition, blood pressure, lipid profile, fasting plasma glucose, glycated haemoglobin (HbA1c), insulin sensitivity, remission of prediabetes, incidence of T2DM, cardiometabolic risk markers, health-related quality of life, physical functioning, treatment adherence and persistence, adverse events, serious adverse events, and treatment discontinuation due to adverse events.

Eligible study designs included Phase III randomised controlled trials, randomised withdrawal trials, long-term extension studies, prespecified secondary or post hoc analyses reporting unique outcomes, and prospective or retrospective real-world observational cohort studies. Narrative reviews, systematic reviews, meta-analyses, conference abstracts, editorials, commentaries, letters without original data, case reports, case series, study protocols, laboratory or animal studies, and non-English publications were excluded. Where multiple publications originated from the same parent trial, each report was assessed individually. Publications reporting unique prespecified outcomes were included in the qualitative synthesis, whereas duplicate reports containing the same participant population and outcome data were excluded to avoid double-counting of evidence.

Two reviewers independently screened titles, abstracts, and full-text articles against the eligibility criteria. Data extraction was performed independently using a standardised data extraction form, and disagreements were resolved through discussion and consensus. Methodological quality was assessed using the Cochrane Risk of Bias 2 (RoB 2) tool for randomised studies and the Risk Of Bias In Non-randomized Studies of Interventions (ROBINS-I) tool for observational studies.

Search Strategy

The electronic search strategy was developed according to recommendations in the Cochrane Handbook for Systematic Reviews of Interventions [22]. Controlled vocabulary terms, including Medical Subject Headings (MeSH) and Emtree terms, were combined with free-text keywords related to tirzepatide, obesity, overweight, weight loss, body mass index, cardiometabolic outcomes, and real-world evidence using the Boolean operators AND and OR. Search strategies were adapted for each database to maximise sensitivity while maintaining specificity. No restrictions were placed on publication year or country of origin; however, only studies published in English were eligible for inclusion.

Owing to substantial clinical and methodological heterogeneity across study populations, interventions, outcome measures, follow-up durations, and study designs, quantitative meta-analysis was not considered appropriate. Instead, findings were synthesised narratively in accordance with the Synthesis Without Meta-analysis (SWiM) reporting guideline. The complete electronic search strategies, including database-specific subject headings, keywords, Boolean operators, and the number of records retrieved from Ovid MEDLINE ALL, Embase, CENTRAL, and CINAHL Ultimate, are presented in Tables 1-4.

**Table 1:** Embase Search Strategy (1974 to June 30, 2026)

Step	Search Query	Results
1	exp Obesity/	842,877
2	(Obesity or Obese or Excess body weight or overweight).mp. [mp=title, abstract, heading word, drug trade name, original title, device manufacturer, drug manufacturer, device trade name, keyword heading word, floating subheading word, candidate term word]	932,302
3	exp Tirzepatide/	5,220
4	(Tirzepatide or Mounjaro or Zepbound or LY3298176 or "GLP-1/GIP Dual Receptor Agonist*").mp. [mp=title, abstract, heading word, drug trade name, original title, device manufacturer, drug manufacturer, device trade name, keyword heading word, floating subheading word, candidate term word]	5,574
5	1 OR 2	997,759
6	3 OR 4	5,574
7	5 AND 6	3,679

**Table 2:** Ovid MEDLINE (ALL) Search Strategy (1946 to June 30, 2026)

Search No.	Search Strategy	Records Retrieved
1	exp Obesity/	299,201
2	(Obesity OR Obese OR "Excess body weight" OR Overweight).mp. [mp=title, book title, abstract, original title, name of substance word, subject heading word, floating sub-heading word, keyword heading word, organism supplementary concept word, protocol supplementary concept word, rare disease supplementary concept word, unique identifier, synonyms, population supplementary concept word, anatomy supplementary concept word]	548,042
3	exp Tirzepatide/	819
4	(Tirzepatide OR Mounjaro OR Zepbound OR LY3298176 OR "GLP-1/GIP Dual Receptor Agonist*").mp. [mp=title, book title, abstract, original title, name of substance word, subject heading word, floating sub-heading word, keyword heading word, organism supplementary concept word, protocol supplementary concept word, rare disease supplementary concept word, unique identifier, synonyms, population supplementary concept word, anatomy supplementary concept word]	2,380
5	1 OR 2	550,427
6	3 OR 4	2,380
7	5 AND 6	1,459

**Table 3:** Cochrane Central Register of Controlled Trials (CENTRAL) Search Strategy (From Inception to June 30, 2026)

Search No.	Search Strategy	Records Retrieved
#1	MeSH descriptor: [Obesity] explode all trees	23,925
#2	Obesity OR Obese OR "Excess body weight" OR Overweight	76,081
#3	MeSH descriptor: [Tirzepatide] explode all trees	287
#4	Tirzepatide OR Mounjaro OR Zepbound OR LY3298176 OR "GLP-1/GIP Dual Receptor Agonist*"	1,061
#5	#1 OR #2	76,189
#6	#3 OR #4	1,061
#7	#5 AND #6	614

**Table 4:** Cumulative Index to Nursing and Allied Health Literature (CINAHL) Search Strategy (From Inception to June 30, 2026)

Search No.	Search Strategy	Records Retrieved
S1	(MH "Obesity+")	124,067
S2	Obesity OR Obese OR "Excess body weight" OR Overweight	180,029
S3	"Tirzepatide"	644
S4	Tirzepatide OR Mounjaro OR Zepbound OR LY3298176 OR "dual GIP:GLP-1 receptor agonist*"	670
S5	S1 OR S2	180,666
S6	S3 OR S4	670
S7	S5 AND S6	325

Study Selection

**Figure 1:**
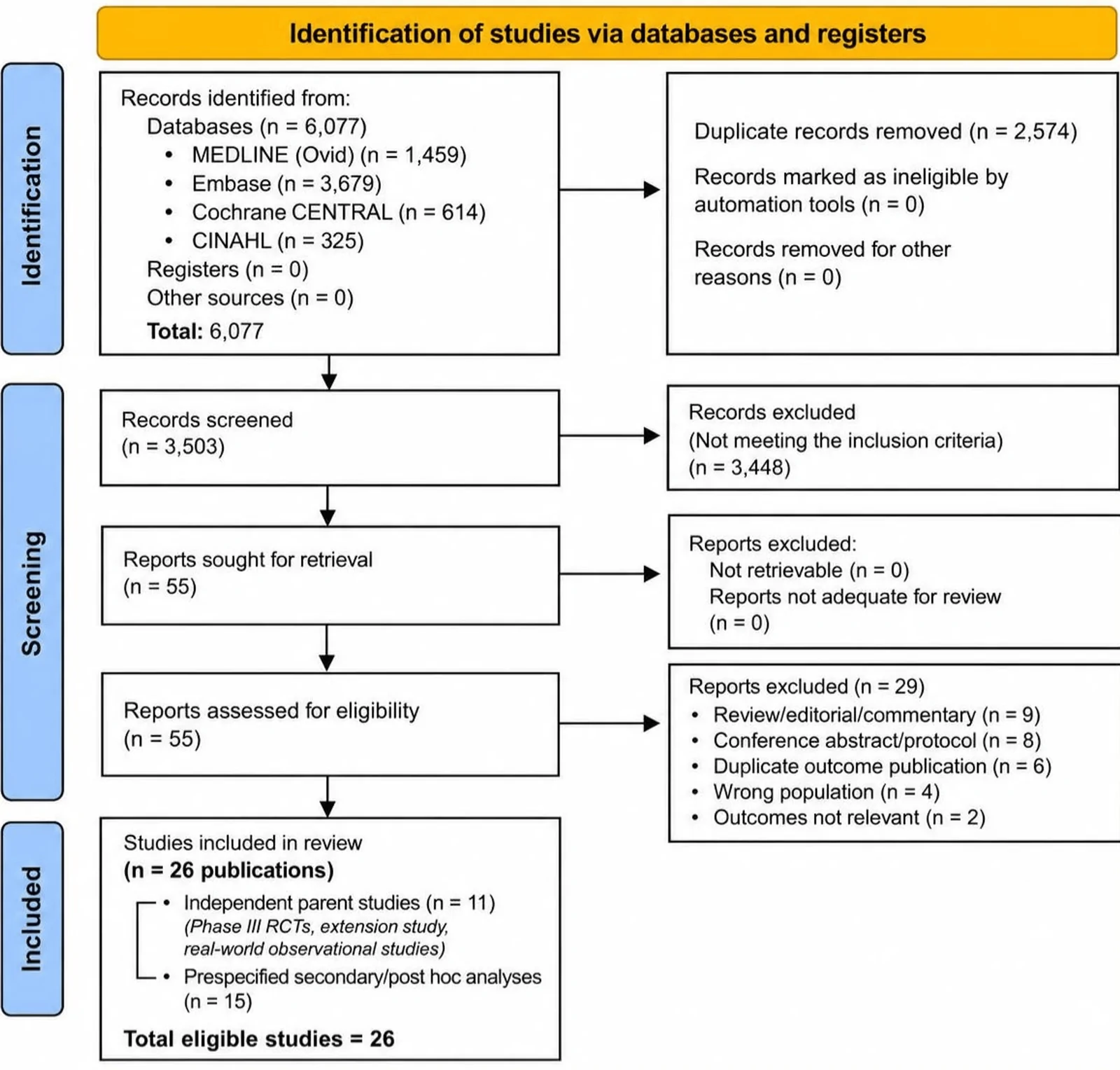
PRISMA 2020 Flow Diagram for the Study Selection Process PRISMA, Preferred Reporting Items for Systematic Reviews and Meta-Analyses.

All records identified through electronic database searches were imported into Rayyan (Rayyan Systems, Inc., Cambridge, Massachusetts) to manage references, remove duplicates, and screen studies [23]. After using both automatic and manual methods to remove duplicates, two reviewers independently screened the titles and abstracts based on predefined eligibility criteria. If either reviewer considered a study potentially eligible, it proceeded to full-text review, which was also conducted independently by the same reviewers. When there were disagreements about a study’s eligibility, the reviewers discussed the issue and reached a consensus. If needed, a third reviewer helped make the final decision. Reasons for excluding studies at the full-text stage were recorded and are shown in the PRISMA 2020 flow diagram [24]. To avoid including the same evidence more than once, multiple publications from the same parent trial were carefully assessed. Publications reporting unique, prespecified outcomes were included in the qualitative synthesis, whereas duplicates reporting the same participants and outcome data were excluded. Figure 1 shows the PRISMA flow diagram.

Data Extraction

For each study, information was collected on study characteristics (first author, year, country, design, parent trial, funding, and setting), participant characteristics (sample size, age, sex, baseline BMI, obesity classification, and relevant comorbidities), and intervention characteristics (tirzepatide dose, comparator, treatment duration, follow-up, lifestyle intervention, and treatment discontinuation). Data were also gathered on efficacy outcomes (changes in body weight, weight-loss maintenance, weight regain after treatment discontinuation, BMI, waist circumference, and body composition), cardiometabolic outcomes (blood pressure, lipid profile, fasting plasma glucose, HbA1c, insulin sensitivity, inflammatory markers, prediabetes remission, type 2 diabetes incidence, and cardiovascular risk markers), patient-reported outcomes (quality of life, physical functioning, treatment adherence, and persistence), and safety outcomes (adverse events, serious adverse events, events leading to treatment discontinuation, and other safety outcomes). Each publication was screened and assessed individually. During data extraction, related publications were linked to identify shared participant cohorts and prevent duplicate extraction. Data were extracted from all eligible publications; however, only outcomes uniquely reported in each secondary publication were included in the synthesis. When identical outcomes were reported in multiple publications, data were taken from the most comprehensive and up-to-date report. This approach ensured a comprehensive synthesis of all relevant evidence while preserving the independence of each parent study and minimising duplicate reporting bias.

Risk of Bias Assessment

The quality of the included studies was independently assessed by two reviewers using validated tools appropriate for each study design. For randomised controlled trials, the RoB 2 tool [25] was applied to evaluate bias in randomisation, deviations from intended interventions, missing data, outcome measurement, and reporting. Each domain was rated as low risk, some concerns, or high risk, with an overall judgement made according to RoB 2 guidelines. Observational studies were assessed using the ROBINS-I tool [26], which evaluates bias due to confounding, participant selection, intervention classification, deviations from intended interventions, missing data, outcome measurement, and reporting. Overall ratings were classified as low, moderate, serious, or critical risk of bias. Disagreements between reviewers were resolved through discussion and, if necessary, consultation with a third reviewer. The risk-of-bias assessments were summarised in both narrative and graphical formats.

Data Synthesis

A quantitative meta-analysis was not conducted due to substantial heterogeneity among the included studies in terms of design and methodology. These differences encompassed study design, participant characteristics, intervention protocols, comparator groups, treatment duration, follow-up periods, and reported outcomes. The evidence base comprised randomised controlled trials, long-term extension studies, prespecified secondary analyses, and real-world observational studies. Consequently, findings were synthesised narratively in accordance with the Synthesis Without Meta-analysis (SWiM) guideline [27]. The evidence was organised into six outcome domains: weight-loss efficacy; weight-loss maintenance and weight regain following treatment discontinuation; cardiometabolic outcomes; patient-reported outcomes; safety and tolerability; and real-world effectiveness. When multiple publications originated from the same parent trial, each was reviewed separately, but only unique outcomes were included to prevent duplication of data. Main trial reports provided primary efficacy and safety data, while secondary analyses contributed additional information on mechanisms, cardiometabolic outcomes, patient-reported outcomes, and subgroup analyses. Real-world observational studies were evaluated separately to assess the generalisability of trial findings to routine clinical practice.

Results

Study Selection

The database search identified 6,077 records: 1,459 from Ovid MEDLINE ALL, 3,679 from Embase, 614 from the Cochrane Central Register of Controlled Trials (CENTRAL), and 325 from CINAHL Ultimate. After removal of 2,574 duplicates, 3,503 unique records were screened by title and abstract. Of these, 3,448 were excluded for failing to meet the eligibility criteria. Full texts of 55 articles were assessed, and 26 publications met the inclusion criteria for qualitative synthesis. These comprised 11 independent parent studies [6,13,15-21,28,29] and 15 prespecified secondary analyses [30-44], encompassing eight Phase III randomised controlled trials, one long-term extension study, two real-world observational cohort studies, and secondary analyses reporting unique efficacy, cardiometabolic, mechanistic, safety, and patient-reported outcomes. The primary reasons for exclusion at the full-text stage were ineligible study designs (including reviews, editorials, conference abstracts, and study protocols), duplicate publications, studies with populations outside the eligibility criteria, and publications lacking relevant outcomes. Due to substantial clinical and methodological heterogeneity, a quantitative meta-analysis was not performed. Instead, results were summarised narratively according to predefined outcome domains. The study selection process is depicted in the PRISMA 2020 flow diagram (Figure 1).

Characteristics of Included Studies

A total of 26 publications from 2022 to 2026 were included in the qualitative synthesis, originating from 11 independent parent studies involving over 30,000 adults with overweight or obesity and 15 prespecified secondary analyses from the SURMOUNT clinical programme. The evidence base comprised eight Phase III randomised controlled trials, one long-term extension study, two real-world observational cohort studies, and 15 prespecified secondary analyses. The randomised controlled trials evaluated once-weekly tirzepatide at doses of 5 mg, 10 mg, or 15 mg in adults with overweight or obesity, with or without comorbid conditions. Comparator groups included placebo, semaglutide 2.4 mg, lifestyle intervention, or treatment withdrawal. Treatment durations ranged from 52 to 88 weeks, with extended follow-up in the SURMOUNT-1 extension study.

The included studies enrolled adults with obesity without diabetes, adults with obesity and type 2 diabetes, individuals with obesity-related sleep apnoea, Chinese adults with obesity, Japanese adults with obesity, and participants who had completed intensive lifestyle interventions. The two real-world observational cohort studies evaluated the effectiveness of tirzepatide in routine clinical practice for adults with overweight or obesity, thereby complementing the evidence from the randomised trials.

The prespecified secondary analyses supplemented the primary efficacy studies by examining body composition, insulin sensitivity, β-cell function, ambulatory blood pressure, cardiovascular risk markers, nutritional status, psychiatric safety, physical functioning, quality of life, predictors of treatment response, and treatment effects in key subgroups. Further details regarding the included studies are provided in Tables 5, 6.

**Table 5:** Characteristics of the 11 Independent Parent Studies RCT, randomised controlled trial; SHAPE, Real-World Weight Loss Observed With Semaglutide and Tirzepatide in Patients with OverweigHt or Obesity And Without TyPE 2 Diabetes.

Author (Year)	Study	Design	Population	Sample Size	Comparator	Duration	Primary Focus
Jastreboff et al. (2022) [15]	SURMOUNT-1	Phase III RCT	Adults with obesity or overweight (≥1 weight-related comorbidity) without diabetes	2,539	Placebo	72 weeks	Weight loss
Garvey et al. (2023) [28]	SURMOUNT-2	Phase III RCT	Adults with obesity or overweight and type 2 diabetes	938	Placebo	72 weeks	Weight loss
Wadden et al. (2023) [6]	SURMOUNT-3	Phase III RCT	Adults with obesity or overweight who achieved ≥5% weight loss during a 12-week intensive lifestyle intervention.	579	Placebo	72 weeks	Additional weight loss following intensive lifestyle intervention
Aronne et al. (2024) [13]	SURMOUNT-4	Randomized withdrawal RCT	Adults with obesity or overweight (without diabetes) after a 36-week open-label tirzepatide lead-in.	783	Withdrawal vs continuation	88 weeks	Maintenance of weight loss
Malhotra et al. (2024) [17]	SURMOUNT-OSA	Phase III RCT	Adults with obesity and moderate-to-severe obstructive sleep apnoea, with and without positive airway pressure therapy.	469 + 465	Placebo	52 weeks	Reduction in OSA severity and body weight
Zhao et al. (2024) [18]	SURMOUNT-CN	Phase III RCT	Chinese adults with obesity or overweight and at least one weight-related comorbidity.	210	Placebo	52 weeks	Weight loss
Kadowaki et al. (2025) [16]	SURMOUNT-J	Phase III RCT	Japanese adults with obesity disease.	399	Placebo	72 weeks	Weight loss
Aronne et al. (2025) [29]	SURMOUNT-5	Phase III RCT	Adults with obesity or overweight with at least one weight-related comorbidity.	751	Semaglutide 2.4 mg once weekly	72 weeks	Head-to-head comparison
Jastreboff et al. (2025) [19]	SURMOUNT-1 Extension	Extension study	Participants from SURMOUNT-1 with obesity or overweight and prediabetes at baseline.	1,032	Continued tirzepatide versus placebo during the parent trial with off-treatment follow-up.	176 weeks	Long-term weight maintenance and prevention of type 2 diabetes
Rodriguez et al. (2024) [21]	Comparative cohort	Retrospective comparative observational cohort.	Adults with overweight or obesity receiving tirzepatide or semaglutide in routine clinical practice	18,386	Semaglutide	Variable	Follow-up to 12 months (or the maximum follow-up reported in the paper
Ng et al. (2025) [20]	SHAPE	Real-world cohort	Obesity/overweight	9,699	Semaglutide	Variable	Real-world effectiveness

**Table 6:** Characteristics of Prespecified Secondary Analyses Included in the Review

Author (Year)	Parent Trial(s)	Publication Type	Primary Outcome Evaluated	Contribution to the Evidence Base
Mari et al. (2025) [31]	SURMOUNT-1	Post hoc analysis	β-cell function, insulin sensitivity, and glucose metabolism	Demonstrated that tirzepatide improved insulin sensitivity and β-cell function beyond the effects expected from weight loss alone, providing mechanistic evidence supporting diabetes prevention
Look et al. (2025) [30]	SURMOUNT-1	Post hoc analysis	Changes in fat mass, lean mass, and body composition	Fat mass reduction
Horn et al. (2025) [32]	SURMOUNT-1 & SURMOUNT-4	Post hoc analysis	Time to weight plateau and predictors of plateau	Characterised the time to weight plateau and identified predictors of a longer time to plateau during tirzepatide treatment, providing insights into the trajectory of long-term weight loss
Ard et al. (2025) [33]	SURMOUNT-1	Post hoc analysis	Early treatment response as a predictor of long-term weight loss	Demonstrated that early weight loss response predicted greater long-term weight reduction, supporting the use of early treatment response as a predictor of long-term treatment success
Krumholz et al. (2024) [34]	SURMOUNT-1	Post hoc analysis	Clinic blood pressure according to baseline blood pressure category	Demonstrated clinically meaningful reductions in systolic and diastolic blood pressure across baseline blood pressure and hypertension categories, supporting the cardiovascular benefits of tirzepatide irrespective of baseline hypertension status
de Lemos et al. (2024) [35]	SURMOUNT-1	ABPM substudy	24-hour ambulatory blood pressure	Demonstrated clinically meaningful reductions in 24-hour ambulatory systolic and diastolic blood pressure, confirming sustained blood pressure lowering under ambulatory monitoring and complementing findings based on clinic blood pressure measurements
Sattar et al. (2026) [36]	SURMOUNT-1	Post hoc analysis	Cardiometabolic and inflammatory biomarkers	Demonstrated broad improvements in biomarkers of inflammation, metabolic and hepatic stress, endothelial dysfunction, and selected hemostatic pathways, providing mechanistic evidence supporting the potential cardiovascular benefits of tirzepatide beyond weight loss
Sattar et al. (2026) [37]	SURMOUNT-1 & SURMOUNT-4	Post hoc analysis	Transitions between BMI categories	Demonstrated that sustained tirzepatide therapy facilitated clinically meaningful transitions to lower BMI categories, with these shifts accompanied by progressive improvements in cardiometabolic risk factors, reinforcing the clinical relevance of durable weight reduction
Wadden et al. (2026) [38]	SURMOUNT-1, SURMOUNT-2, & SURMOUNT-3	Post hoc analysis	Psychiatric safety	Provided comprehensive evidence that tirzepatide was not associated with an increased risk of psychiatric adverse events across pooled Phase III SURMOUNT trials, supporting its favourable neuropsychiatric safety profile in adults with obesity without known major psychopathology
Li et al. (2026) [39]	SURMOUNT-1, SURMOUNT-3, & SURMOUNT-4	Post hoc analysis	Physical functioning	Demonstrated clinically meaningful improvements in patient-reported physical functioning across the SURMOUNT programme, with the greatest benefits observed among participants with the greatest baseline functional impairment, supporting both weight loss-dependent and weight loss-independent mechanisms of functional improvement
Gourgari et al. (2025) [40]	SURMOUNT-1, SURMOUNT-3, & SURMOUNT-4	Post hoc analysis	Treatment response according to age of obesity onset	Demonstrated that tirzepatide produced comparable weight loss and cardiometabolic improvements regardless of age at obesity onset, supporting its efficacy in adults with both early- and later-onset obesity despite differences in baseline metabolic characteristics
Kokkinos et al. (2026) [41]	SURMOUNT-1 & SURMOUNT-2	Post hoc analysis	Early treatment response	Demonstrated that early weight-loss responders achieved greater long-term weight reduction and cardiometabolic improvements while maintaining a comparable gastrointestinal tolerability profile, supporting early treatment response as a clinically useful predictor of therapeutic benefit
Galindo et al. (2026) [42]	SURMOUNT-1, SURMOUNT-3, & SURMOUNT-4	Post hoc analysis	Concomitant medications associated with weight gain	Demonstrated that tirzepatide produced clinically meaningful and sustained weight loss despite the concomitant use of medications associated with weight gain, supporting its effectiveness in patients with complex treatment regimens encountered in routine clinical practice
Almandoz et al. (2026) [43]	SURMOUNT-1, SURMOUNT-2, SURMOUNT-3, & SURMOUNT-4	Post hoc analysis	Nutritional biomarkers and dietary adequacy during prolonged treatment	Provided comprehensive evidence that prolonged tirzepatide treatment was not associated with clinically meaningful macronutrient malnutrition or increased vitamin deficiency-related adverse events, supporting the overall nutritional safety of tirzepatide while highlighting the importance of routine nutritional monitoring during obesity management
Gudzune et al. (2025) [44]	SURMOUNT-1	Post hoc analysis	Obesity-specific health-related quality of life	Demonstrated that greater weight reduction with tirzepatide was associated with progressive, clinically meaningful improvements in obesity-specific and generic health-related quality of life, with the largest benefits observed among participants achieving the greatest weight loss and those with baseline physical or psychosocial limitations

Weight-Loss Efficacy

Randomised controlled trials reported dose-dependent weight loss with tirzepatide in adults with overweight or obesity, including participants with type 2 diabetes, obesity-related obstructive sleep apnoea (OSA), and Asian populations.

In the SURMOUNT-1 trial, Jastreboff et al. [15] reported mean percentage weight reductions of 15.0%, 19.5%, and 20.9% in the 5 mg, 10 mg, and 15 mg tirzepatide groups, respectively, compared with 3.1% in the placebo group after 72 weeks. More than half of participants receiving the 10 mg and 15 mg doses achieved at least 20% body weight reduction. Reductions in body mass index (BMI) and waist circumference were also reported.

In the SURMOUNT-2 trial, Garvey et al. [28] reported mean weight reductions approaching 15% among adults with obesity and type 2 diabetes receiving tirzepatide. Improvements in glycated haemoglobin (HbA1c) and fasting plasma glucose were also reported relative to placebo.

Following an intensive lifestyle intervention, participants in the SURMOUNT-3 trial who received tirzepatide achieved an additional mean weight reduction of 18.4%, whereas participants randomised to placebo regained weight during follow-up [6].

In the SURMOUNT-CN and SURMOUNT-J trials, Zhao et al. and Kadowaki et al. reported reductions in body weight across all approved tirzepatide doses compared with placebo in Chinese and Japanese adults, respectively [16,18].

The SURMOUNT-5 trial directly compared tirzepatide with semaglutide 2.4 mg. Aronne et al. [29] reported greater mean percentage weight reduction with tirzepatide than with semaglutide, together with higher proportions of participants achieving weight-loss thresholds of ≥10%, ≥15%, ≥20%, and ≥25%.

Prespecified secondary analyses reported additional weight-related outcomes. Look et al. [30] reported reductions in fat mass with relative preservation of lean body mass. Horn et al. [32] reported that body weight reached a plateau following prolonged treatment. Ard et al. [33] reported higher proportions of participants maintaining long-term weight loss among those who achieved an early treatment response. Additional subgroup analyses reported weight-loss outcomes among participants with early-onset obesity [40], those receiving medications associated with weight gain [42], and participants who achieved greater initial weight reduction [41].

Three real-world observational cohort studies [20,21,43] reported sustained reductions in body weight in routine clinical practice over follow-up periods of 12 to 24 months.

Detailed weight-loss outcomes from randomised controlled trials, prespecified secondary analyses, and real-world observational studies are presented in Table 7.

**Table 7:** Summary of Weight-Loss Efficacy Across the Included Studies T2DM, type 2 diabetes mellitus.

Author (Year)	Study	Population	Treatment Duration	Key Weight-Loss Findings
Jastreboff et al. (2022) [15]	SURMOUNT-1	Obesity without diabetes	72 weeks	Dose-dependent weight reductions of 15.0%–20.9%; >50% achieved ≥20% weight loss
Garvey et al. (2023) [28]	SURMOUNT-2	Obesity with T2DM	72 weeks	Significant weight loss with concurrent glycaemic improvement
Wadden et al. (2023) [6]	SURMOUNT-3	Post-lifestyle intervention	72 weeks	Additional 18.4% weight reduction following intensive lifestyle intervention
Zhao et al. (2024) [18]	SURMOUNT-CN	Chinese adults	52 weeks	Clinically meaningful placebo-adjusted weight reduction
Kadowaki et al. (2025) [16]	SURMOUNT-J	Japanese adults	72 weeks	Consistent efficacy across all tirzepatide doses
Aronne et al. (2025) [29]	SURMOUNT-5	Obesity/overweight	72 weeks	Greater weight loss than semaglutide; more participants achieved ≥10–25% weight-loss thresholds
Look et al. (2025) [30]	SURMOUNT-1	Secondary analysis	72 weeks	Weight reduction primarily attributable to fat mass loss
Ard et al. (2025) [33]	SURMOUNT-1	Secondary analysis	72 weeks	Early weight loss predicted long-term success
Horn et al. (2025) [32]	SURMOUNT-1/4	Secondary analysis	Long-term	Defined timing of weight-loss plateau
Gourgari et al. (2025) [40]	SURMOUNT programme	Secondary analysis	Variable	Consistent efficacy in early-onset obesity
Galindo et al. (2026) [42]	SURMOUNT programme	Secondary analysis	Variable	Sustained efficacy despite concomitant weight-inducing medications
Kokkinos et al. (2026) [41]	SURMOUNT-1/2	Secondary analysis	Variable	Early responders achieved greater long-term weight loss

Weight-Loss Maintenance and Weight Regain Following Treatment Discontinuation

Weight-loss maintenance and weight regain following treatment discontinuation were evaluated in the SURMOUNT-4 randomised withdrawal trial, the SURMOUNT-1 extension study, and several prespecified secondary analyses.

In the SURMOUNT-4 trial, participants received open-label tirzepatide for 36 weeks before being randomised to continue tirzepatide or switch to placebo for an additional 52 weeks [13]. Participants who continued tirzepatide achieved a further 5.5% reduction in body weight, whereas those switched to placebo regained approximately 14.0% of body weight during follow-up. The cumulative mean weight reduction from baseline was approximately 25% among participants who continued tirzepatide. Participants remaining on tirzepatide were also more likely to maintain ≥80% of their initial weight loss than those receiving placebo [13].

The SURMOUNT-1 extension study reported sustained reductions in body weight throughout the extended treatment period [19]. During the off-treatment follow-up period, participants who discontinued tirzepatide experienced progressive weight regain, although mean body weight remained below baseline values. The extension study also reported a lower incidence of progression from prediabetes to type 2 diabetes among participants receiving tirzepatide than among those receiving placebo [19].

Prespecified secondary analyses reported additional findings related to long-term weight-loss trajectories. Horn et al. [32] reported that body weight reached a plateau following prolonged treatment. Participants receiving higher maintenance doses and those achieving greater early weight reduction reached this plateau later than participants with smaller initial responses. Ard et al. [33] reported higher rates of long-term weight-loss maintenance among participants who achieved greater weight reduction during the early stages of treatment.

Subgroup analyses also reported long-term weight-loss outcomes in specific patient populations. Kokkinos et al. [41] reported sustained weight reduction among participants who achieved greater early weight-loss responses, whereas Galindo et al. [42] reported sustained weight loss among participants receiving concomitant medications associated with weight gain.

Weight-loss maintenance and weight regain following treatment discontinuation across the included studies are summarised in Table 8.

**Table 8:** Weight-Loss Maintenance and Weight Regain Following Treatment Discontinuation T2DM, type 2 diabetes mellitus.

Author (Year)	Study	Outcome Evaluated	Key Findings	Clinical Implications
Aronne et al. (2024) [13]	SURMOUNT-4	Weight-loss maintenance	Continued tirzepatide produced additional weight loss, whereas placebo withdrawal resulted in substantial weight regain	Long-term treatment is required to maintain weight reduction
Jastreboff et al. (2025) [19]	SURMOUNT-1 Extension	Long-term weight maintenance and diabetes prevention	Sustained weight reduction during treatment with marked reduction in incident T2DM; progressive weight regain after discontinuation	Supports prolonged treatment for durable metabolic benefit
Horn et al. (2025) [32]	SURMOUNT-1 & SURMOUNT-4	Weight-loss plateau	Weight loss stabilised after prolonged treatment; plateau occurred later among participants with greater early response	Defines expected long-term treatment trajectory
Ard et al. (2025)[33]	SURMOUNT-1	Early response	Early weight loss predicted long-term success	Early treatment response may guide prognosis
Kokkinos et al. (2026) [41]	SURMOUNT-1 & SURMOUNT-2	Early responders	Greater early response associated with superior long-term efficacy without reduced tolerability	Supports individualized treatment expectations
Galindo et al. (2026) [42]	SURMOUNT programme	Concomitant medications	Sustained weight loss despite use of weight-inducing medications	Demonstrates efficacy across complex clinical populations

Cardiometabolic Outcomes

Cardiometabolic outcomes were reported across randomised controlled trials, long-term extension studies, and prespecified secondary analyses. Reported outcomes included glycaemic measures, blood pressure, insulin sensitivity, β-cell function, lipid parameters, inflammatory biomarkers, incidence of type 2 diabetes, and physical functioning.

In the SURMOUNT-1 trial, Jastreboff et al. [15] reported reductions in waist circumference, systolic and diastolic blood pressure, fasting plasma glucose, fasting insulin, and lipid levels among participants receiving tirzepatide compared with placebo. The SURMOUNT-CN and SURMOUNT-J trials also reported reductions in these cardiometabolic measures among Chinese and Japanese adults, respectively [16,18].

In the SURMOUNT-2 trial, Garvey et al. [28] reported reductions in glycated haemoglobin (HbA1c), fasting plasma glucose, and body weight among participants with obesity and type 2 diabetes receiving tirzepatide compared with placebo.

The SURMOUNT-1 extension study evaluated cardiometabolic outcomes during longer-term follow-up. Jastreboff et al. [19] reported maintenance of glycaemic status and a lower incidence of progression from prediabetes to type 2 diabetes among participants receiving tirzepatide than among those receiving placebo.

Prespecified secondary analyses reported additional metabolic outcomes. Mari et al. [31] reported changes in insulin sensitivity and β-cell function among participants with overweight or obesity and either prediabetes or normoglycaemia.

Blood pressure outcomes were reported in dedicated secondary analyses. Krumholz et al. [34] reported reductions in clinic systolic and diastolic blood pressure across baseline blood pressure subgroups. De Lemos et al. [35] reported reductions in 24-hour ambulatory systolic and diastolic blood pressure during both daytime and nighttime monitoring.

Additional secondary analyses evaluated cardiovascular risk markers. Sattar et al. [36] reported changes in inflammatory biomarkers, lipid parameters, and other cardiometabolic risk markers following tirzepatide treatment. Another prespecified analysis [37] reported changes in cardiometabolic risk factors among participants who transitioned to lower BMI categories during treatment.

Physical functioning outcomes were reported by Li et al. [39], who evaluated changes in physical functioning scores during tirzepatide treatment across baseline physical functioning categories.

Cardiometabolic outcomes reported across randomised controlled trials, long-term extension studies, and prespecified secondary analyses are summarised in Table 9.

**Table 9:** Cardiometabolic Outcomes Associated With Tirzepatide Treatment T2DM, type 2 diabetes mellitus.

Author (Year)	Study	Outcome Domain	Key Findings	Clinical Significance
Jastreboff et al. (2022) [15]	SURMOUNT-1	Cardiometabolic profile	Reduced waist circumference, blood pressure, fasting glucose, and lipid levels	Demonstrated broad metabolic improvements accompanying weight loss
Garvey et al. (2023) [28]	SURMOUNT-2	Glycaemic control	Significant reductions in HbA1c and fasting plasma glucose	Effective in obesity with T2DM
Zhao et al. (2024) [18]	SURMOUNT-CN	Metabolic outcomes	Consistent improvements in cardiometabolic parameters	Confirmed efficacy in Chinese adults
Kadowaki et al. (2025) [16]	SURMOUNT-J	Metabolic outcomes	Similar improvements observed in Japanese adults	Demonstrated generalizability across Asian populations
Jastreboff et al. (2025) [19]	SURMOUNT-1 Extension	Diabetes prevention	Markedly reduced progression from prediabetes to T2DM	Supports long-term disease modification
Mari et al. (2025) [31]	SURMOUNT-1	β-cell function and insulin sensitivity	Improved insulin sensitivity and β-cell function	Provides mechanistic explanation for glycaemic improvement
Krumholz et al. (2024) [34]	SURMOUNT-1	Clinic blood pressure	Significant reductions in systolic and diastolic blood pressure	Reduced cardiovascular risk factors
de Lemos et al. (2024) [35]	SURMOUNT-1	Ambulatory blood pressure	Sustained reductions in 24-hour blood pressure	Confirmed blood pressure improvement using ABPM
Sattar et al. (2026) [36]	SURMOUNT-1	Cardiovascular biomarkers	Improved inflammatory and metabolic biomarkers	Suggests comprehensive cardiometabolic risk reduction
Sattar et al. (2026) [37]	SURMOUNT-1 & SURMOUNT-4	BMI transitions	Lower BMI categories associated with improved cardiometabolic profile	Reinforces relationship between weight loss and metabolic health
Li et al. (2026) [39]	SURMOUNT programme	Physical functioning	Greatest improvements among participants with poor baseline function	Demonstrates translation of metabolic improvements into functional benefit

Patient-Reported Outcomes and Functional Benefits

Patient-reported outcomes were evaluated in several prespecified secondary analyses of the SURMOUNT clinical programme. Reported outcomes included health-related quality of life (HRQoL), physical functioning, psychiatric safety, and nutritional status.

In a prespecified analysis of SURMOUNT-1, Gudzune et al. [44] reported greater improvements in obesity-specific HRQoL among participants receiving tirzepatide than among those receiving placebo. Reported domains included physical functioning, self-esteem, sexual well-being, public distress, and work-related functioning.

Physical functioning outcomes were reported by Li et al. [39], who evaluated changes in mobility, physical activity, activities of daily living, and physical functioning scores during tirzepatide treatment across baseline functional status categories.

Psychiatric safety was assessed in a post hoc analysis of the SURMOUNT programme. Wadden et al. [38] reported the incidence of depression, anxiety, suicidal ideation, and other psychiatric adverse events among participants receiving tirzepatide and placebo. The incidence of these events was similar across treatment groups.

Nutritional status during treatment was evaluated by Almandoz et al. [43] using data from the SURMOUNT-1, SURMOUNT-3, and SURMOUNT-4 trials. Reported outcomes included nutritional biomarkers measured during treatment together with changes in body weight and energy intake.

Patient-reported outcomes and functional measures reported across the included studies are summarised in Table 10.

**Table 10:** Patient-Reported Outcomes and Functional Benefits of Tirzepatide

Author (Year)	Parent Trial(s)	Outcome Domain	Key Findings	Clinical Relevance
Gudzune et al. (2025) [44]	SURMOUNT-1	Health-related quality of life	Significant improvements in obesity-specific quality of life, including physical function, self-esteem, work productivity, and social functioning	Demonstrates that substantial weight loss translates into meaningful improvements in patients' daily lives
Li et al. (2026) [39]	SURMOUNT programme	Physical functioning	Greatest improvements observed among participants with the poorest baseline physical function	Supports improved mobility, independence, and functional capacity
Wadden et al. (2026) [38]	SURMOUNT programme	Psychiatric safety	No increased risk of depression, anxiety, suicidal ideation, or major psychiatric adverse events	Supports the neuropsychiatric safety of long-term tirzepatide treatment
Almandoz et al. (2026) [43]	SURMOUNT-1, SURMOUNT-3, SURMOUNT-4	Nutritional status	Clinically meaningful weight loss occurred without evidence of widespread nutritional compromise	Reassures clinicians regarding nutritional safety during intensive weight reduction

Safety and Tolerability

Safety and tolerability outcomes were reported across the randomised controlled trials, long-term extension studies, and prespecified secondary analyses. Gastrointestinal adverse events were the most frequently reported treatment-emergent adverse events and were generally mild to moderate in severity.

In SURMOUNT-1, Jastreboff et al. [15] reported nausea, diarrhoea, vomiting, and constipation as the most common adverse events, with incidence increasing modestly at higher tirzepatide doses. Most events occurred during the dose-escalation phase, were transient, and resolved without treatment discontinuation. Similar safety findings were reported in SURMOUNT-2 [28], SURMOUNT-3 [6], SURMOUNT-4 [13], SURMOUNT-CN [18], and SURMOUNT-J [16], demonstrating a consistent safety profile across different clinical populations and ethnic groups.

Long-term safety was evaluated in the SURMOUNT-4 randomised withdrawal trial, in which continued tirzepatide treatment for up to 88 weeks was not associated with new safety signals, and the overall adverse event profile remained consistent with that observed during earlier treatment phases [13].

The head-to-head SURMOUNT-5 trial further evaluated safety relative to semaglutide 2.4 mg. Aronne et al. [29] reported a higher incidence of gastrointestinal adverse events with tirzepatide, whereas the incidence of serious adverse events and treatment discontinuation due to adverse events was low and comparable between treatment groups.

Psychiatric safety was examined in a prespecified post hoc analysis of the SURMOUNT programme. Wadden et al. [38] reported no increased incidence of depression, anxiety, suicidal ideation, or other major psychiatric adverse events among participants receiving tirzepatide compared with placebo, with overall psychiatric event rates remaining low across treatment groups.

Nutritional safety was evaluated by Almandoz et al. [43] using data from the SURMOUNT-1, SURMOUNT-3, and SURMOUNT-4 trials. Despite substantial reductions in body weight and energy intake, nutritional biomarkers generally remained within acceptable ranges throughout treatment.

Additional subgroup analyses demonstrated consistent safety findings across clinically relevant populations. Galindo et al. [42] reported that concomitant use of medications associated with weight gain did not adversely affect the tolerability of tirzepatide, while Kokkinos et al. [41] found no increase in adverse events among participants achieving greater early weight loss.

Across the included studies, serious adverse events were uncommon and occurred at similar frequencies in tirzepatide and comparator groups. Most treatment discontinuations were attributable to gastrointestinal symptoms during dose escalation rather than serious adverse events. No consistent increase in pancreatitis, medullary thyroid carcinoma, severe hypoglycaemia among participants without diabetes, or other major safety concerns was reported during the available follow-up. Detailed safety and tolerability outcomes are summarised in Table 11.

**Table 11:** Safety and Tolerability Outcomes of Tirzepatide T2DM, type 2 diabetes mellitus.

Author (Year)	Study	Safety Domain	Key Findings	Clinical Implications
Jastreboff et al. (2022) [15]	SURMOUNT-1	Overall safety	Gastrointestinal adverse events were the most common; predominantly mild to moderate and transient	Established the initial safety profile of tirzepatide
Garvey et al. (2023) [28]	SURMOUNT-2	Safety in T2DM	Similar gastrointestinal safety profile observed in participants with obesity and T2DM	Demonstrated consistent tolerability in diabetes
Wadden et al. (2023) [6]	SURMOUNT-3	Long-term tolerability	Adverse events remained consistent following intensive lifestyle intervention	Confirmed tolerability after lifestyle modification
Aronne et al. (2024) [13]	SURMOUNT-4	Long-term safety	No new safety signals during continued treatment over 88 weeks	Supports prolonged treatment for chronic obesity management
Zhao et al. (2024) [18]	SURMOUNT-CN	Ethnic subgroup safety	Comparable adverse-event profile in Chinese adults	Demonstrated consistency across populations
Kadowaki et al. (2025) [16]	SURMOUNT-J	Ethnic subgroup safety	Similar safety profile in Japanese adults	Supports generalizability of safety findings
Aronne et al. (2025) [29]	SURMOUNT-5	Comparative safety	Greater efficacy than semaglutide with comparable rates of serious adverse events	Confirms favourable benefit-risk balance
Wadden et al. (2026) [38]	SURMOUNT programme	Psychiatric safety	No increased risk of depression, anxiety, or suicidal ideation	Reassures clinicians regarding neuropsychiatric safety
Almandoz et al. (2026) [43]	SURMOUNT-1/3/4	Nutritional safety	No clinically significant deterioration in nutritional status	Supports nutritional safety during substantial weight loss
Galindo et al. (2026) [42]	SURMOUNT programme	Medication interactions	Weight-inducing medications did not adversely affect tolerability	Supports use in patients receiving concomitant therapies
Kokkinos et al. (2026) [41]	SURMOUNT-1/2	Early treatment response	Greater early weight loss was not associated with increased adverse events	Supports individualized treatment without compromising safety

Real-World Effectiveness

Two real-world observational cohort studies evaluated tirzepatide in adults with overweight or obesity receiving treatment in routine clinical practice.

Rodriguez et al. [21] conducted a comparative cohort study of adults with overweight or obesity receiving either tirzepatide or semaglutide. Participants receiving tirzepatide achieved greater reductions in body weight, and higher proportions reached weight-loss thresholds of ≥5%, ≥10%, and ≥15% of baseline body weight than participants receiving semaglutide.

Ng et al. [20] reported findings from the SHAPE (Real-World Weight Loss Observed With Semaglutide and Tirzepatide in Patients with OverweigHt or Obesity And Without TyPE 2 Diabetes) observational cohort involving adults with overweight or obesity without type 2 diabetes. Reductions in body weight were reported throughout the follow-up period among participants receiving tirzepatide in routine clinical practice. Weight-loss outcomes reported in the real-world observational studies are summarised in Table 11.

Certainty of the Evidence (GRADE)

The certainty of the evidence was evaluated using the Grading of Recommendations Assessment, Development and Evaluation (GRADE) framework. Randomised controlled trials were initially classified as high-certainty evidence, while observational studies were classified as low-certainty evidence. Certainty ratings were then adjusted based on factors such as risk of bias, inconsistency, indirectness, imprecision, publication bias, magnitude of effect, dose-response relationship, and the potential influence of residual confounding on the observed treatment effect. The overall certainty of evidence for each outcome was categorised as high, moderate, low, or very low, following established GRADE guidance [45,46].

Table 12 presents the certainty of the evidence for each outcome. Most outcomes demonstrated moderate to high certainty, with the strongest evidence supporting weight-loss effectiveness, maintenance of weight loss during treatment, and overall safety.

**Table 12:** GRADE Assessment of the Certainty of Evidence GRADE, Grading of Recommendations Assessment, Development and Evaluation; RCT, randomised controlled trial.

Outcome	Study Design	Risk of Bias	Inconsistency	Indirectness	Imprecision	Publication Bias	Certainty of Evidence
Weight-loss efficacy	Phase III RCTs	Not serious	Not serious	Not serious	Not serious	Undetected	High ⨁⨁⨁⨁
Weight-loss maintenance	RCT + Extension study	Not serious	Not serious	Not serious	Not serious	Undetected	High ⨁⨁⨁⨁
Weight regain after discontinuation	Randomised withdrawal trial	Not serious	Not serious	Not serious	Not serious	Undetected	High ⨁⨁⨁⨁
Cardiometabolic outcomes	RCTs + secondary analyses	Not serious	Not serious	Not serious	Serious	Undetected	Moderate ⨁⨁⨁○
Diabetes prevention	Extension study	Not serious	Not serious	Not serious	Serious	Undetected	Moderate ⨁⨁⨁○
Patient-reported outcomes	Secondary analyses	Serious	Not serious	Not serious	Not serious	Undetected	Moderate ⨁⨁⨁○
Safety and tolerability	Phase III RCTs	Not serious	Not serious	Not serious	Not serious	Undetected	High ⨁⨁⨁⨁
Real-world effectiveness	Observational studies	Serious	Not serious	Not serious	Not serious	Undetected	Moderate ⨁⨁⨁○

Strong evidence was observed for the primary outcomes of weight-loss effectiveness, maintenance of weight loss during ongoing treatment, and overall safety, based on consistent findings from multiple large Phase III trials. For cardiometabolic outcomes and diabetes prevention, the certainty of evidence was moderate to high, supported by similar results in randomised trials and planned secondary analyses, although some findings were derived from secondary rather than primary trial endpoints.

The certainty of evidence for patient-reported outcomes was moderate, primarily because these results were derived from a limited number of planned secondary analyses. The evidence for real-world effectiveness was also moderate, as it was based on observational studies, despite their findings being consistent with those from randomised trials.

In summary, the GRADE assessment indicates that tirzepatide is effective and generally well tolerated in adults with overweight or obesity. There is strong evidence supporting its effects on weight loss, maintenance of weight loss, and safety, as well as moderate to high certainty regarding its broader benefits for cardiometabolic health and patient-centred outcomes.

Risk of Bias Assessment

The methodological quality of the included studies was assessed independently by two reviewers using validated tools appropriate to each study design. Randomised controlled trials were evaluated using the Cochrane RoB 2 tool, whereas non-randomised studies, including the long-term extension study and real-world observational cohort studies, were assessed using the ROBINS-I tool [22,25,26]. Any disagreements between reviewers were resolved through discussion and consensus in accordance with the recommendations of the Cochrane Handbook for Systematic Reviews of Interventions [22].

Overall, the randomised evidence was judged to be of high methodological quality, with a low overall risk of bias across most domains (Table 13). All Phase III SURMOUNT trials employed centralised computer-generated randomisation, allocation concealment, double-blinding of participants, investigators, and outcome assessors, together with standardised outcome assessment procedures [6,13,15-18,28,29]. Objective outcome measures, including body weight, body mass index, glycated haemoglobin, blood pressure, waist circumference, and laboratory biomarkers, minimised the risk of measurement bias. Attrition rates were generally low and balanced between treatment groups, and intention-to-treat analyses with appropriate handling of missing outcome data were consistently applied [6,13,15-18,28,29]. The SURMOUNT-4 randomised withdrawal trial was judged to have some concerns regarding missing outcome data because only participants who completed the open-label lead-in phase underwent randomisation, introducing a potential risk of attrition-related bias [13].

The non-randomised studies were judged to have an overall moderate risk of bias according to the ROBINS-I tool (Table 14). The SURMOUNT-1 long-term extension study and the two real-world observational cohort studies were primarily limited by the potential for residual confounding, participant selection, and loss to follow-up, which are inherent limitations of non-randomised study designs [19-21,26]. Nevertheless, all three studies demonstrated a low risk of bias in the classification of interventions, outcome measurement, handling of missing data, and selection of the reported results, supporting the overall reliability of their findings.

The 15 prespecified secondary analyses originated from the included parent studies and therefore inherited the corresponding methodological quality assessments. In accordance with Cochrane guidance, these secondary publications were not assessed independently because they analysed the same underlying trial populations while reporting unique prespecified outcomes [22]. The overall risk-of-bias assessments are presented in Tables 13, 14.

**Table 13:** Risk of Bias Assessment of Included Randomized Controlled Trials Using the Cochrane Risk of Bias 2 (RoB 2) Tool RoB 2, Cochrane Risk of Bias 2; RCT, randomised controlled trial.

Parent Study	Study Design	Randomization Process	Deviations From Intended Interventions	Missing Outcome Data	Measurement of the Outcome	Selection of the Reported Result	Overall Risk of Bias
Jastreboff et al. (2022) (SURMOUNT-1) [15]	Phase III double-blind RCT	Low	Low	Low	Low	Low	Low
Garvey et al. (2023) (SURMOUNT-2) [28]	Phase III double-blind RCT	Low	Low	Low	Low	Low	Low
Wadden et al. (2023) (SURMOUNT-3) [6]	Phase III double-blind RCT	Low	Low	Low	Low	Low	Low
Aronne et al. (2024) (SURMOUNT-4) [13]	Randomized withdrawal Phase III RCT	Low	Low	Low	Low	Low	Low
Malhotra et al. (2024) (SURMOUNT-OSA) [17]	Two parallel Phase III RCTs	Low	Low	Low	Low	Low	Low
Zhao et al. (2024) (SURMOUNT-CN) [18]	Phase III double-blind RCT	Low	Low	Low	Low	Low	Low
Kadowaki et al. (2025) (SURMOUNT-J) [16]	Phase III double-blind RCT	Low	Low	Low	Low	Low	Low
Aronne et al. (2025) (SURMOUNT-5) [29]	Phase III active-comparator RCT	Low	Low	Low	Low	Low	Low

**Table 14:** Risk of Bias Assessment of Included Non-randomized Studies Using the ROBINS-I Tool ROBINS-I, Risk Of Bias In Non-randomized Studies of Interventions.

Parent Study	Study Design	Bias Due to Confounding	Bias in Selection of Participants	Bias in Classification of Interventions	Bias Due to Deviations From Intended Interventions	Bias Due to Missing Data	Bias in Measurement of Outcomes	Bias in Selection of the Reported Result	Overall Risk of Bias
Jastreboff et al. (2025) (SURMOUNT-1 long-term extension) [19]	Long-term extension study	Moderate	Low	Low	Low	Low	Low	Low	Moderate
Rodriguez et al. (2025) [21]	Real-world retrospective observational cohort	Moderate	Moderate	Low	Low	Low	Low	Low	Moderate
Ng et al. (2026) (SHAPE Study) [20]	Real-world retrospective observational cohort	Moderate	Moderate	Low	Low	Low	Low	Low	Moderate

Discussion

This systematic review synthesised the most recent evidence on the long-term efficacy, safety, weight-loss maintenance, weight regain following treatment discontinuation, cardiometabolic outcomes, patient-reported outcomes, and real-world effectiveness of tirzepatide in adults with overweight or obesity. The review included 26 publications representing 11 independent parent studies and 15 prespecified secondary analyses, encompassing Phase III randomised controlled trials, long-term extension studies, a randomised withdrawal trial, mechanistic analyses, and real-world observational cohorts. By integrating evidence from these complementary study designs, this review provides a comprehensive assessment of the long-term therapeutic profile of tirzepatide.

Compared with previous systematic reviews, which primarily evaluated the initial SURMOUNT Phase III trials and focused on short-term efficacy and safety, the present review incorporates more recent evidence from long-term follow-up studies, prespecified secondary analyses, and routine clinical practice. This broader evidence base enabled evaluation of treatment durability, weight regain following treatment discontinuation, changes in body composition, cardiometabolic risk factors, insulin sensitivity, β-cell function, blood pressure, nutritional status, psychiatric safety, physical functioning, and health-related quality of life. The integration of evidence from randomised trials and real-world studies is consistent with contemporary approaches to evidence synthesis that support clinical decision-making, guideline development, and healthcare policy [19-44,47-49].

Across the included studies, tirzepatide was consistently associated with substantial reductions in body weight, together with improvements in multiple cardiometabolic outcomes, physical functioning, and obesity-specific quality of life. However, findings from the SURMOUNT-4 randomised withdrawal trial and the SURMOUNT-1 extension study indicated that discontinuation of treatment was followed by progressive weight regain, whereas continued treatment was associated with maintenance of weight loss and sustained metabolic benefits [13,19]. These observations are consistent with the chronic and relapsing nature of obesity and highlight the importance of long-term management strategies.

The findings of this review also support the growing recognition of obesity as a chronic, progressive, and relapsing metabolic disease rather than simply a condition characterised by excess body weight. Recent international clinical practice guidelines recommend a complications-centred approach to obesity management, in which treatment success is assessed not only by weight reduction but also by improvements in obesity-related comorbidities, metabolic health, physical functioning, and patient-reported outcomes [50-52]. The outcomes reported across the included studies are consistent with this multidimensional approach, with improvements observed in glycaemic control, blood pressure, lipid parameters, physical functioning, and health-related quality of life, in addition to reductions in body weight.

The observation that weight regain occurred following treatment discontinuation has important implications for clinical practice. Similar to the long-term management of hypertension, dyslipidaemia, and type 2 diabetes mellitus, obesity pharmacotherapy may require ongoing treatment for many individuals to maintain therapeutic benefits. Decisions regarding treatment duration should nevertheless remain individualised, taking into account clinical response, adverse events, patient preferences, treatment costs, and shared decision-making between patients and healthcare professionals.

Biological Mechanisms Underlying the Long-Term Clinical Effects of Tirzepatide

**Figure 2:**
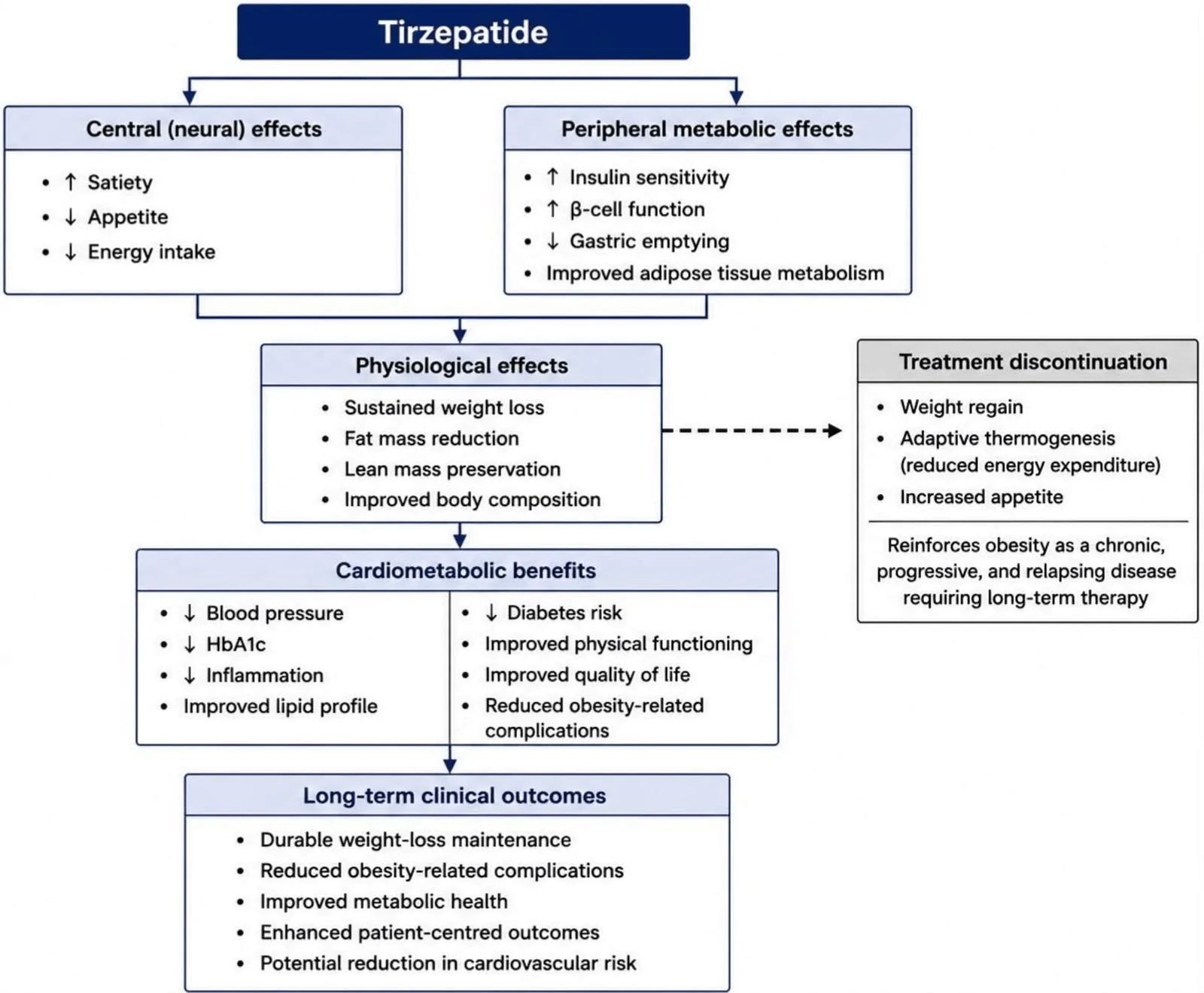
Proposed Mechanisms Underlying the Long-Term Clinical Benefits of Tirzepatide in Adults With Overweight or Obesity Dual GIP/GLP-1 receptor agonism by tirzepatide promotes sustained weight loss through complementary central and peripheral metabolic effects, resulting in improved body composition and cardiometabolic health. Treatment discontinuation may lead to adaptive physiological responses and weight regain, supporting the need for long-term obesity management. GIP, glucose-dependent insulinotropic polypeptide; GLP-1, glucagon-like peptide-1; ↑, increase; ↓, decrease. Image credits: Created by Aliyu O. Olaniyi and Happiness Olaniyi.

The biological mechanisms underlying the long-term clinical benefits of tirzepatide are illustrated in Figure 2. As a dual glucose-dependent insulinotropic polypeptide (GIP) and glucagon-like peptide-1 (GLP-1) receptor agonist, tirzepatide exerts coordinated effects on central appetite regulation and peripheral metabolic pathways. Central nervous system actions enhance satiety and reduce energy intake, while peripheral effects improve insulin sensitivity, preserve pancreatic β-cell function, delay gastric emptying, and favourably modulate adipose tissue metabolism. Collectively, these complementary mechanisms promote sustained reductions in body weight and fat mass, relative preservation of lean body mass, and favourable improvements in body composition, culminating in broad cardiometabolic benefits. Figure 2 also illustrates the adaptive physiological responses associated with treatment discontinuation, including increased appetite, adaptive reductions in energy expenditure, and progressive weight regain. These physiological adaptations provide a biological explanation for the chronic, progressive, and relapsing nature of obesity and underscore the need for sustained long-term pharmacotherapy to maintain therapeutic benefits.

The favourable clinical outcomes observed across the SURMOUNT programme are biologically plausible given tirzepatide's dual agonist activity at both the GIP and GLP-1 receptors. Simultaneous activation of these incretin pathways influences appetite regulation, energy homeostasis, glucose metabolism, and adipose tissue function. Compared with selective GLP-1 receptor agonists, tirzepatide has been shown to enhance satiety, reduce energy intake, delay gastric emptying, improve insulin sensitivity, preserve β-cell function, and favourably influence lipid metabolism, thereby contributing to greater reductions in body weight and improvements in cardiometabolic risk factors [10-12,53-55]. The findings of the SURMOUNT-5 trial are consistent with these mechanisms, with tirzepatide producing greater weight loss than semaglutide 2.4 mg in a head-to-head comparison [29].

Beyond weight reduction, this review demonstrates that tirzepatide is associated with improvements across multiple cardiometabolic outcomes. The included studies reported favourable changes in insulin sensitivity, β-cell function, blood pressure, inflammatory biomarkers, lipid parameters, and progression from prediabetes to type 2 diabetes [19,31,34-37]. These findings suggest that the metabolic effects of tirzepatide extend beyond weight reduction alone. Secondary analyses also reported preferential reductions in fat mass while preserving lean body mass, together with improvements in inflammatory and cardiovascular risk markers [30,31,36]. These observations are consistent with current obesity management guidelines, which emphasise improvements in metabolic health, physical functioning, and obesity-related complications, in addition to weight reduction, as important indicators of treatment success [50-52].

The present review also indicates that the therapeutic effects of tirzepatide were reported across a broad range of patient populations. Beneficial outcomes were observed among adults with obesity, participants with type 2 diabetes, individuals with obesity-related obstructive sleep apnoea, Chinese and Japanese populations, and participants undergoing intensive lifestyle intervention [6,15-18,28]. Prespecified subgroup analyses reported similar treatment effects among participants with early-onset obesity, those receiving medications associated with weight gain, and those with varying baseline cardiometabolic risk profiles [40-42]. In addition, the real-world observational studies included in this review reported weight-loss outcomes that were broadly similar to those observed in the Phase III clinical trials [20,21]. Although observational studies are inherently more susceptible to bias than randomised controlled trials, these findings provide supportive evidence that the effectiveness of tirzepatide extends beyond controlled clinical trial settings.

Clinical Perspective and Implications for Practice

The findings of this review have important implications for the contemporary management of obesity and reinforce the evolving paradigm that obesity should be recognised and treated as a chronic, progressive, and relapsing metabolic disease rather than simply a consequence of excess body weight. Historically, obesity management has emphasised short-term weight reduction through lifestyle modification, with pharmacotherapy frequently regarded as a temporary adjunct and often discontinued once an initial weight-loss target had been achieved. However, the evidence synthesised in the present review challenges this traditional approach by consistently demonstrating that sustained pharmacological therapy is required to maintain clinically meaningful weight reduction and its associated cardiometabolic benefits. The substantial weight regain observed following treatment discontinuation in the SURMOUNT-4 randomised withdrawal trial and the SURMOUNT-1 extension study reflects persistent biological adaptations favouring restoration of body weight, including increased appetite, adaptive reductions in energy expenditure, and neuroendocrine mechanisms that defend adiposity [13,19,56-58]. Consequently, discontinuation of effective pharmacotherapy should not necessarily be interpreted as treatment success but rather as withdrawal of therapy for a chronic disease, analogous to discontinuing antihypertensive, lipid-lowering, or glucose-lowering therapy after achieving therapeutic targets. These observations are consistent with contemporary international obesity guidelines, which increasingly advocate long-term pharmacological treatment for appropriately selected individuals to prevent disease progression, reduce obesity-related complications, and maintain durable clinical benefit [50-52,59,60].

Importantly, the therapeutic benefits of tirzepatide extend well beyond reductions in body weight alone. Improvements in glycaemic control, insulin sensitivity, blood pressure, inflammatory biomarkers, body composition, physical functioning, health-related quality of life, and overall cardiometabolic risk demonstrate that treatment success should be evaluated using a multidimensional framework rather than changes in body weight or body mass index alone [19,30-42]. This holistic approach reflects the increasing shift towards complications-centred obesity management, in which the principal objective is improvement in obesity-related morbidity, functional status, and patient-centred outcomes rather than achievement of an arbitrary weight-loss target [50-52]. Furthermore, preservation of lean body mass during substantial reductions in fat mass supports the clinical value of tirzepatide by demonstrating favourable improvements in body composition rather than indiscriminate weight loss [30]. Collectively, these findings suggest that tirzepatide has the potential to modify the natural history of obesity by simultaneously improving multiple interrelated metabolic abnormalities that contribute to long-term cardiovascular and metabolic risk.

The head-to-head superiority of tirzepatide over semaglutide 2.4 mg, as demonstrated in the SURMOUNT-5 trial, has important implications for therapeutic decision-making in the rapidly evolving landscape of obesity pharmacotherapy [29]. Although GLP-1 receptor agonists have transformed medical obesity management during the past decade, the greater and more sustained weight reduction achieved through dual GIP/GLP-1 receptor agonism suggests that tirzepatide may establish a new benchmark for pharmacological obesity treatment in appropriately selected patients. Nevertheless, treatment selection should remain individualised and should incorporate patient preferences, obesity-related complications, comorbidities, treatment tolerability, contraindications, accessibility, and cost alongside anticipated clinical benefit. As additional incretin-based therapies continue to emerge, comparative effectiveness studies evaluating long-term clinical outcomes, treatment persistence, quality of life, cardiovascular benefits, and cost-effectiveness will be essential for defining the optimal sequencing and selection of anti-obesity pharmacotherapies [57,58].

Another important contribution of this review is the inclusion of emerging real-world observational evidence, which complements findings from randomised controlled trials and enhances confidence in the generalisability of the available data. Although randomised trials remain the gold standard for establishing efficacy, their findings may not always reflect routine clinical practice because of strict eligibility criteria, intensive follow-up, and highly selected patient populations. The available observational studies demonstrated weight-loss outcomes and safety profiles that were broadly consistent with those reported across the SURMOUNT programme [20,21], suggesting that the substantial clinical benefits observed under trial conditions are reproducible within everyday healthcare settings. Nevertheless, long-term treatment persistence, medication adherence, equitable access to therapy, and healthcare resource availability remain important determinants of real-world effectiveness and should be considered when implementing pharmacotherapy within routine obesity services.

From a healthcare systems perspective, the increasing availability of highly effective anti-obesity pharmacotherapy has the potential to substantially reduce the burden of obesity-related complications, including type 2 diabetes mellitus, hypertension, cardiovascular disease, obstructive sleep apnoea, metabolic dysfunction-associated steatotic liver disease, chronic kidney disease, and other obesity-associated conditions [15-19,31-37]. However, widespread implementation will require careful consideration of healthcare resources, equitable access, long-term treatment adherence, and cost-effectiveness. Although formal economic evaluation was beyond the scope of the present review, previous work has emphasised the importance of integrating robust clinical evidence with pharmacoeconomic analyses when evaluating innovative therapeutic interventions to ensure that clinical effectiveness is translated into sustainable healthcare policy and practice [47]. Future health-economic studies should therefore determine whether the higher acquisition costs of tirzepatide are offset by reductions in obesity-related complications, hospital admissions, healthcare utilisation, productivity losses, and improvements in quality-adjusted life years.

Finally, the findings of this review support the ongoing transition towards personalised obesity management. Prespecified subgroup analyses demonstrated consistent efficacy across individuals with and without type 2 diabetes mellitus, different ethnic populations, varying ages of obesity onset, and patients receiving concomitant weight-promoting medications [6,15-18,40-42]. Nevertheless, further research is required to identify clinical, metabolic, genetic, and molecular predictors of treatment response that may facilitate precision medicine approaches. Integration of biomarkers, digital health technologies, and artificial intelligence into routine obesity care may enable more individualised treatment selection, improve long-term treatment adherence, optimise healthcare resource utilisation, and maximise patient-centred outcomes. As the therapeutic landscape continues to evolve, precision obesity medicine is likely to become an increasingly important component of long-term obesity management.

Strengths and Limitations

This systematic review demonstrates several key strengths that enhance its scientific value and clinical relevance. It represents one of the most comprehensive evaluations of the long-term effects, safety, weight-loss maintenance, weight regain following treatment discontinuation, cardiometabolic outcomes, patient-reported outcomes, and real-world effectiveness of tirzepatide in adults with overweight or obesity. In contrast to earlier reviews that primarily examined the initial SURMOUNT trials, this review incorporates recent long-term extension studies, randomised withdrawal trials, planned secondary analyses, and new real-world observational studies. This approach provides a more complete understanding of treatment durability and broader clinical effects [19-44]. By integrating mechanistic studies, body composition data, cardiometabolic outcomes, quality-of-life measures, and real-world evidence, the review provides a comprehensive overview of current research and addresses the need for a multidimensional evaluation of anti-obesity therapies.

The review's methodology is another notable strength. It adhered to PRISMA 2020 guidelines, established explicit eligibility criteria using the PICOS framework, conducted comprehensive searches across multiple electronic databases, and employed independent reviewers for study selection and data extraction. Study quality was assessed using the Cochrane Risk of Bias 2 tool for randomised trials and the ROBINS-I tool for non-randomised studies. The certainty of evidence was evaluated using the GRADE framework, and the narrative synthesis followed the SWiM reporting guideline [22-27,53]. Collectively, these methodological approaches enhance the transparency and reliability of the review and its alignment with international standards for systematic reviews [47-49].

The review addressed multiple publications from the same clinical trials with methodological rigour. Rather than excluding all secondary publications, it included planned post hoc analyses only when they reported novel clinical outcomes absent from the primary trial publication. This strategy enabled a comprehensive assessment of mechanistic, cardiometabolic, functional, nutritional, and patient-reported outcomes without duplicating participant data. The inclusion of real-world observational studies further corroborated findings from randomised trials, thereby increasing the relevance and applicability of the evidence to routine clinical practice [20,21,43,44].

Despite these strengths, several limitations should be acknowledged. First, a quantitative meta-analysis was not conducted due to substantial heterogeneity among the included studies in terms of populations, treatment durations, comparator groups, outcomes, and study designs. Consequently, while the narrative synthesis facilitated a broad evaluation of the evidence, it precluded the pooling of results and formal assessment of statistical differences or publication bias. Second, a significant proportion of the studies originated from the SURMOUNT programme and involved overlapping participant groups. Although the review included only unique outcomes from each publication, the majority of evidence is derived from a limited number of large trials. Third, most randomised trials and some secondary analyses received industry funding, which may introduce sponsorship bias despite the generally low risk of bias. Finally, although emerging real-world evidence supports the effectiveness and safety of tirzepatide, these observational studies remain limited in number, duration, and geographic diversity. Additional independent, long-term studies involving diverse populations are necessary to further elucidate treatment persistence, effectiveness, cardiovascular outcomes, rare adverse events, and safety beyond current trials.

In summary, the strengths of this review substantially outweigh its limitations. By synthesising evidence from high-quality randomised trials, long-term extension studies, planned secondary analyses, and recent real-world observational studies through a rigorous review process, this work offers one of the most comprehensive and clinically informative evaluations of tirzepatide to date. The findings establish a robust foundation for clinical decision-making, future research, and the advancement of evidence-based obesity care.

Future Directions

The SURMOUNT clinical programme has demonstrated substantial progress; however, critical questions remain regarding the long-term use of tirzepatide for obesity management. Current evidence indicates that tirzepatide produces sustained weight loss, improves cardiovascular and metabolic health, and maintains a favourable safety profile for up to three years. Nevertheless, the durability of these benefits beyond five years remains uncertain. Future long-term studies should assess treatment adherence, persistence of weight loss, effects on cardiovascular and renal health, long-term safety, and therapeutic effectiveness across diverse patient populations. Such evidence will clarify whether the improvements observed in clinical trials translate into sustained reductions in obesity-related morbidity, mortality, and healthcare utilisation.

Future research should aim to identify predictors of individual treatment response, thereby facilitating more personalised approaches to obesity management. Evidence indicates that tirzepatide is effective in individuals both with and without type 2 diabetes, across various ethnicities and ages of obesity onset, and among those using weight-promoting medications [6,15-18,40-42]. However, variability in treatment response persists. Integrating data on clinical characteristics, metabolic profiles, genetics, and behavioural factors and leveraging digital health technologies may enable clinicians to tailor therapies, enhance adherence, and optimise outcomes while efficiently utilising healthcare resources. Advances in artificial intelligence and data analytics may further support personalised decision-making and the development of improved anti-obesity therapies.

Comparative studies evaluating tirzepatide against emerging anti-obesity agents and established interventions such as bariatric surgery are also warranted. The SURMOUNT-5 trial demonstrated that tirzepatide resulted in greater weight loss than semaglutide 2.4 mg [29]; however, additional head-to-head comparisons with other novel pharmacotherapies, combination regimens, and integrated medical-surgical approaches are necessary. These investigations should extend beyond weight loss to include cardiovascular outcomes, treatment persistence, patient-reported experiences, quality of life, cost-effectiveness, and long-term safety. Such comprehensive data will inform clinical and policy decision-making.

As more research becomes available, it is important to examine how tirzepatide affects other health problems linked to obesity. Early studies suggest it may help with sleep apnoea, fatty liver disease, cardiovascular risk factors, and the development of type 2 diabetes [15-19,31-37]. However, more long-term studies are needed to determine whether these benefits translate into fewer serious cardiovascular events, slower kidney disease progression, better liver health, fewer hospital stays, less disability, and a lower risk of early death. These outcomes are now seen as better measures of long-term success than weight loss alone.

Future investigations should also address the sustainable integration of effective anti-obesity pharmacotherapies into routine healthcare delivery. Previous research has demonstrated that establishing efficacy is insufficient; considerations of cost, accessibility, and long-term value are equally important [47]. As global demand for pharmacological obesity treatments increases, studies evaluating cost-effectiveness, treatment adherence, equity of access, and optimal care delivery models will be critical for informing policy and ensuring broad availability of these therapies.

In summary, future research should not only establish the efficacy of tirzepatide but also determine optimal strategies for its long-term use in obesity management. Integrating investigations of pharmacological mechanisms, comparative effectiveness, real-world implementation, personalised medicine approaches, and economic analyses will be essential to maximising benefits for patients and public health.

## Conclusions

Current evidence demonstrates that tirzepatide induces significant and sustained weight loss in adults with overweight or obesity. Additionally, tirzepatide improves cardiometabolic health, body composition, physical function, quality of life, and metabolic risk factors. These benefits are largely maintained with continued therapy, reinforcing the understanding that obesity is a chronic, progressive disease requiring ongoing management rather than short-term interventions. This review synthesises data from major randomised controlled trials, long-term studies, secondary analyses, and recent real-world evidence to provide a comprehensive assessment of tirzepatide. The findings indicate that tirzepatide is an effective long-term therapeutic option for selected adults with overweight or obesity and provide clinicians with current evidence to inform clinical decision-making. Continued research on long-term outcomes, comparative effectiveness, cardiovascular benefits, and real-world application will further elucidate tirzepatide’s role in obesity management.
